# In Vitro and In Vivo Inhibitory Activity of Limonene against Different Isolates of *Candida* spp.

**DOI:** 10.3390/jof6030183

**Published:** 2020-09-22

**Authors:** Julián E. Muñoz, Diego C. P. Rossi, Daniela L. Jabes, David Aciole Barbosa, Fernanda F. M. Cunha, Luiz R. Nunes, Denise C. Arruda, Carlos Pelleschi Taborda

**Affiliations:** 1 Studies in Translational Microbiology and Emerging Diseases Research Group (MICROS), School of Medicine and Health Sciences, Universidad del Rosario, Bogotá D.C 111221, Colombia; juliane.munoz@urosario.edu.co; 2 Department of Microbiology, Biomedical Sciences Institute, University of São Paulo (USP), São Paulo 05508-060, Brazil; rossido@ucmail.uc.edu; 3 Núcleo Integrado de Biotecnologia, Universidade de Mogi das Cruzes (UMC), Mogi das Cruzes-SP 08780-911, Brazil; danielajabes@umc.br (D.L.J.); aciole.d@gmail.com (D.A.B.); cunha.ffernandes@gmail.com (F.F.M.C.); denisearruda@umc.br (D.C.A.); 4 Centro de Ciências Naturais e Humanas, Universidade Federal do ABC (UFABC), São Bernardo do Campo 09210-580, Brazil; luiz.nunes@ufabc.edu.br; 5 Laboratory of Medical Mycology, Institute of Tropical Medicine of São Paulo-LIM53/Medical School, University of São Paulo (USP), São Paulo 05403-000, Brazil

**Keywords:** Candidiasis, *Candida* spp., limonene, treatment, intravaginal infection

## Abstract

Commensal yeast from the genus *Candida* is part of the healthy human microbiota. In some cases, *Candida* spp. dysbiosis can result in candidiasis, the symptoms of which may vary from mild localized rashes to severe disseminated infections. The most prevalent treatments against candidiasis involve fluconazole, itraconazole, miconazole, and caspofungin. Moreover, amphotericin B associated with prolonged azole administration is utilized to control severe cases. Currently, numerous guidelines recommend echinocandins to treat invasive candidiasis. However, resistance to these antifungal drugs has increased dramatically over recent years. Considering this situation, new therapeutic alternatives should be studied to control candidiasis, which has become a major medical concern. Limonene belongs to the group of terpene molecules, known for their pharmacological properties. In this study, we evaluated in vitro the limonene concentration capable of inhibiting the growth of yeast from the genus *Candida* susceptible or resistant to antifungal drugs and its capacity to induce fungal damage. In addition, intravaginal fungal infection assays using a murine model infected by *Candida albicans* were carried out and the fungal burden, histopathology, and scanning electron microscopy were evaluated. All of our results suggest that limonene may play a protective role against the infection process by yeast from the genus *Candida.*

## 1. Introduction

Candidiasis, caused by the opportunistic fungus *Candida albicans*, is one of the most prevalent infections that affect humans and can display severe morbidity in immunosuppressed individuals [1]. In fact, hospitalized patients infected with HIV/AIDS (human immunodeficiency virus/Acquired immunodeficiency syndrome) or chronic obstructive pulmonary disease or undergoing chemotherapy treatments and transplant patients represent the most relevant groups at risk of developing Oropharyngeal Candidiasis (OPC) [2,3]. 

Candidiasis treatment involves different types of traditional antifungals but can be hindered by the emergence of drug-resistance strains of *C. albicans*. This fact has stimulated researchers to develop new therapeutic alternatives against candidiasis [4].

Limonene belongs to the group of terpenes, which is a group of molecules widely present in essential oils from different species of plants (particularly in their flowers and fruit). Many of these compounds have been shown to display antibacterial, antiprotozoal, and antitumor activity, both in vitro and in vivo [5,6,7]. Farnesol, a sesquiterpene, has been shown to inhibit the growth of *C. albicans* yeasts by interfering in the Ras1-cAMP pathway [8], while thymol—a monoterpene—has been shown to induce morphological changes in this fungus [9].

Limonene is also a monoterpene, found in a wide variety of plants, especially in citrus fruits, such as lemons and oranges. Original studies aimed at characterizing the pharmacological properties of this molecule described as R-(+)-limonene, such as antitumoral, antiparasitary, and antifungal activities. At a 5% concentration, limonene was effective in reducing the average size and number of breast carcinomas in an experimental model using laboratory rats [10]. Subsequently, several studies demonstrated the promising effect of R-(+)-limonene against different tumor cells, such as human lung cancer [11], prostate cancer [12], breast cancer [13], and the lymphoma cell line [14]. Limonene also induces apoptosis in gastric cancer and inhibits liver and peritoneal metastasis [15].

Additionally, some authors have demonstrated that limonene can exert a protective effect against diabetes in a treatment experimental model using rats [16]. Moreover, limonene increases the permeation of other drugs, both in in vitro and in vivo models [17,18].

The antimicrobial effects of limonene also involve a variety of mechanisms, as has been verified in *Plasmodium falciparum.* In fact, in vitro assays involving this parasite showed that limonene could inhibit protein isoprenylation, as well as the biosynthesis of some ubiquinones. Limonene has also been tested in combination with other antimalarial drugs, such as fosmidomycin, with promising results for the development of a new strategy of malaria treatment [19]. Arruda and collaborators (2009) showed that limonene is effective at reducing the size of ulcerative lesions in mice experimentally infected with *Leishmania amazonensis* after both topic and intrarectal treatment. Moreover, they revealed the low toxicity of limonene in mammalian cell lines, which was even lower than that observed for the control *Leishmania amazonensis.* The CC50 values (concentrations that killed 50% of mammalian cells) for the cell lines HEK-293 (Human Embryonic Kidney cells) and LLC-MK2 (Rhesus monkey epithelial kidney cells) were higher than 1.012 µM, while the concentration that killed 50% of the parasites (EC50) was 252 µM [20].

Furthermore, limonene seems to induce apoptosis in *Trypanosoma cruzi*, accompanied by fragmentation of the parasite’s DNA [21]. This terpene can similarly modulate specific aspects of the immune response in treated animals, including an increase in nitric oxide production in peritoneal macrophages of BALB/c mice [14,22,23].

Therefore, given the great antimicrobial potential of this monoterpene, the present work aims to evaluate both the in vitro and in vivo effects of limonene against different *Candida* spp. strains. 

## 2. Material and Methods

### 2.1. Animals

Isogenic female BALB/c mice (six animals per group) that were six to eight weeks old were housed in polypropylene cages under Specific-Pathogen-Free (SPF) conditions with a sawdust substrate. Food and water were provided ad libitum and all mice environments were sterilized. Animals used in this study were bred at the University of São Paulo animal facility. All experiments involving animals were conducted and approved by the Ethics Committee of the University of São Paulo (No. 042-127-02) and were conducted in accordance with international recommendations.

To set up the vulvovaginal candidiasis model, it was necessary to induce the female mice to a pseudo-oestrus phase by the subcutaneous administration of 0.5 mg of 17 beta-estradiol valerate (Sigma Chemicals, St Louis, MO, USA) dissolved in sesame oil (Sigma Chemicals, St Louis, MO, USA) three days before vaginal infection, as described by Hamad et al. [24].

### 2.2. Candida albicans Strains 

We used *Candida albicans* ATCC 90028, *Candida krusei* ATCC 6558, *Candida glabrata* ATCC 2001, *Candida parapsilosis* ATCC 22019, and 10 *C. albicans* clinical isolates from patients with recurrent vulvovaginal candidiasis provided by the strain bank of the MICROS group, Universidad del Rosario, Colombia. Fungal cells were cultivated in modified Yeast Extract–Peptone–Dextrose (YPD) medium (1% yeast extract, 2% casein peptone, 2% glucose, pH 6.5) at 36 °C (yeast phase), and liquid cultures were kept at 120 rpm in an orbital shaker.

### 2.3. Antimicrobial Compounds

(R)-(+)-limonene was purchased from Sigma Aldrich (St. Louis, MO, USA) and dissolved in dimethyl sulfoxide (DMSO) for in vitro experiments. To carry out in vivo assays, we prepared a 10% (308.28 µM) limonene topical formulation, adding the compound to commercial neutral cream (10% Wax self-nonionic emulsifier, 2% mineral oil, 5% propylene glycol, and 84% distilled water). Fluconazole (Pfizer Inc., New York, NY, USA) was dissolved in PBS solution to obtain a concentration of 10 mg/kg and then mixed with the same neutral cream described above.

### 2.4. Conditions of Limonene Exposure for Growth Inhibition Assays

Limonene was added to a 30 mL suspension of *C. albicans* and non-*albicans* yeast cultures (OD_600_ = 0.4) at final concentrations of 25, 50, 250, and 500 µM (1% DMSO concentration). Cultures were then incubated under the conditions described above, for 12 h, and fungal growth was monitored hourly, through OD_600_ measurements. All experiments were performed in triplicate. 

### 2.5. MTT Assay

The viability of *C. albicans* (1 × 10^6^) after treatment with limonene was tested in vitro by cultivating cells in increasing concentrations of limonene (0 µM, 25 µM, 50 µM, 100 µM, 250 µM, 500 µM, and 600 µM) for 8 and 17 h. The cell viability was determined by measuring the cleavage of 3(4,5-dimethylthiazol-2-yl)-2,5-diphenyltetrazolium bromide (MTT; Sigma Aldrich, St. Louis, MO, USA). After incubation with limonene, 50 µL of MTT solution (5 mg/mL) was added and cultures were further incubated, at 37 °C, for 5 h. Next, samples were centrifuged at 10.000 rpm for two minutes, the MTT solution was carefully removed, and 100 µL of dimethyl sulfoxide (DMSO) was added to solubilize the produced formazan, prior to absorbance measurements at 570 nm. Assays were performed in triplicate, and results are expressed as the mean percent reduction of cells, compared to untreated controls. 

### 2.6. Intravaginal Infection of BALB/c Mice

The *C. albicans* ATCC 90028 virulent strain was grown in brain heart infusion (Hi-Media Laboratories, Mumbai, India), at 37 °C, for 24 h, under 200 rpm agitation. Afterward, yeasts were centrifugated, washed in PBS (pH 7.2), and analyzed to determine the cell viability. Six animals per group were inoculated intravaginally with a maximum volume of 10 µL of 3 × 10^5^ yeast cells, as described by Muñoz et al. (2017) [25].

### 2.7. Assay for Colony-Forming Units (CFUs)

BALB/c female mice were infected intravaginally with the virulent *C. albicans* strain ATCC 90028 (3 × 10^5^ yeast cells) and submitted to limonene or fluconazole treatment 24 h post-infection. Treatment was continuous for seven days, every 24 h. On the 8th day, mice were sacrificed, and the vaginal canal was removed, weighed, and homogenized in PBS. A 100 µL sample of this suspension was plated on solid brain heart infusion medium (BHI), supplemented with 10 IU/mL streptomycin/penicillin (Cultilab, São Paulo, Brazil), and 500 µg/mL cycloheximide (Sigma, St. Louis, MO, USA). The petri dishes were then incubated at 37 °C, for 24 h, and colonies were counted (1 colony = 1 CFU).

### 2.8. Histopathology

The vaginal canal was removed and fixed in 10% neutral buffered formalin for subsequent paraffin embedding. Sections (5 mm thick) were stained with silver (Grocott stain) and examined microscopically at 100× magnification (Optiphot-2, Nikon, Tokyo, Japan).

### 2.9. Transmission Electron Microscopy (TEM)

*C. albicans* ATCC 90028 yeasts untreated (control group) or treated with 500 µM limonene for 24 h at 37 °C, in RPMI 1640 and buffered with 0.16 M MOPS were harvested in log phase and fixed at room temperature, for two hours, with 2.5% glutaraldehyde and 4% paraformaldehyde in PBS. After this process, yeasts were incubated in 1% osmium for 2 h, dehydrated, and embedded in araldite-Epon, as previously described [26]. Ultrathin sections were collected on grids stained with alcoholic 1% uranyl acetate and lead citrate. The grids were analyzed using a Jeol 100 CX (Tokyo, Japan) transmission electron microscope. 

### 2.10. Scanning Electron Microscopy (SEM)

Vaginal canals from untreated or limonene-treated mice were excised and then fixed in 2.5% glutaraldehyde, for 1 h, at room temperature. Afterwards, samples were transferred to a critical point drier (BAL-TEC CPD-030—Electron Microscopy Sciences, Buffalo Grove, IL, USA). Sample assembly was conducted as previously described [26] and observed using an EM Jeol 100 CX transmission electron microscope.

### 2.11. Statistical Analysis

**Figure 1:**
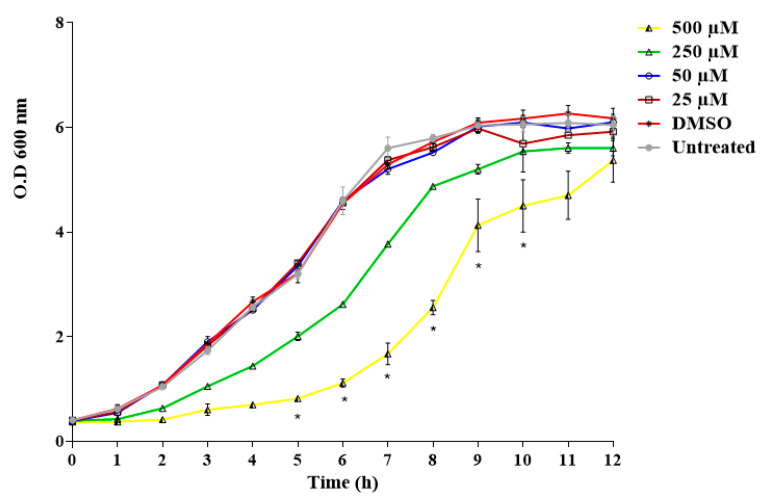
*Candida albicans* ATCC 90028 growth curve in the presence of 0 μM, 25 μM, 50 μM, 250 μM, and 500 μM of limonene. Culture growth was monitored hourly, through OD_600_ readings. Measurements were performed in triplicate, and the graph shows the average values and their respective standard deviations. * *p* < 0.05 compared with the control group (untreated; ANOVA with post-hoc Tukey’s test).

Statistical analysis was conducted using GraphPad Prism, version 7.05 software (GraphPad Software, San Diego, CA, USA). Results were expressed as means ± the standard deviations (SD) of the indicated numbers of animals or experiments. Statistical comparisons were conducted by analysis of variance (one-way ANOVA) followed by nonparametric Tukey’s test. *p* Values of ≤0.05 and ≤0.01 indicated statistical significance. Conditions of limonene exposure for the growth inhibition assay were performed in triplicate, and the graphs show the average values and their respective standard deviations. Figure 1 shows the under-curve area values of the growth patterns of the different strains exposed or not exposed to the limonene treatment for 12 h. The one-way ANOVA was carried out followed by a nonparametric test.

## 3. Results

### 3.1. Evaluation of the Antifungal Activity of Limonene In Vitro

**Figure 2:**
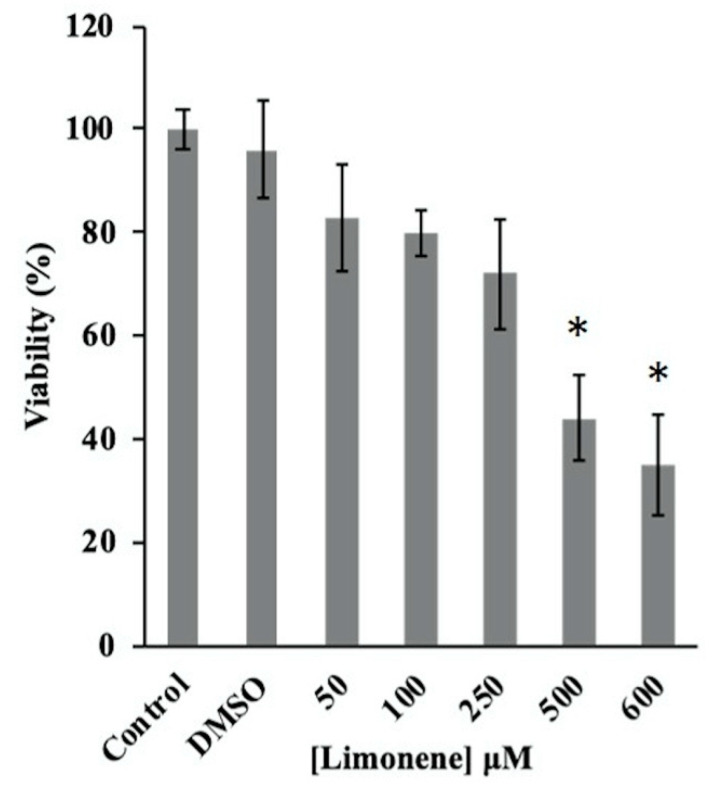
Cell viability of *C. albicans* was evaluated after 8 h of incubation with 50, 100, 250, 500, and 600 µM of limonene. The growth of treated cultures is shown as the percentage of control cells incubated in medium alone. Viability was determined by a 3(4,5-dimethylthiazol-2-yl)-2,5-diphenyltetrazolium bromide (MTT) assay. * *p* < 0.05 compared with the control group (*C. albicans* ATCC 90028 without treatment; ANOVA with post-hoc Tukey’s test).

The time-dependent growth of *C. albicans* ATCC 90028, exposed to different concentrations of limonene (0 μM, 25 μM, 50 μM, 250 μM, and 500 μM), for a period of 12 h, showed inhibition at concentrations of 250 and 500 μM (Figure 1). Moreover, we observed a significant decrease in the cell viability of *C. albicans* when yeasts were previously treated with limonene, at 500 μM and 600 μM concentrations, for a period of 8 h (Figure 2). The calculated EC50% was 444 ± 35 μM. 

**Figure 3:**
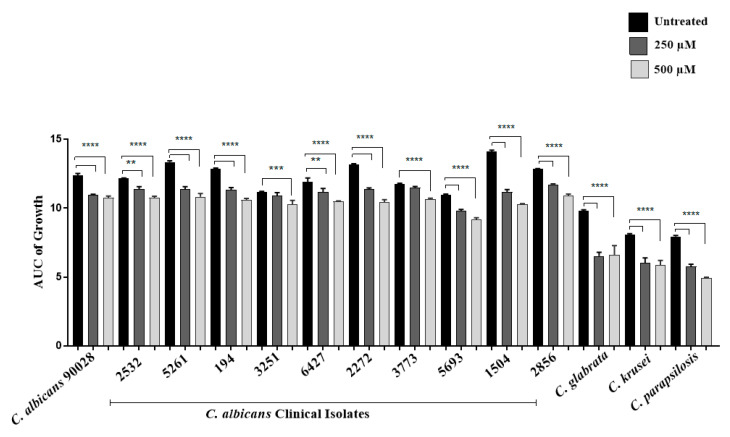
Limonene antifungal activity in ATCC strains of *C. albicans*, *Candida krusei, Candida glabrata, Candida parapsilosis*, and 10 *C. albicans* clinical isolates. Culture growth was monitored hourly, through OD_600_ readings. Measurements were performed in triplicate, and the graph shows the areas under the curve and their respective standard deviations. ** *p* < 0.01, *** *p* < 0.001, and **** *p* < 0.0001 compared with the control group (untreated; ANOVA with post-hoc Tukey’s test).

Limonene antifungal activity using 250 and 500 µM was analyzed in ATCC strains of *C. albicans*, *C. krusei*, *C. glabrata*, *C. parapsilosis*, and in 10 additional *C. albicans* clinical isolates. In the case of the three non-*albicans* species, a significant growth decrease was observed compared to the control group that did not receive limonene treatment. The clinical strains (*n* = 10) analyzed showed a less marked, but statistically significant, growth decrease, as shown in Figure 3.

### 3.2. Treatment with Limonene Reduces the Fungal Burden in Mice with Vaginal Candidiasis

**Figure 4:**
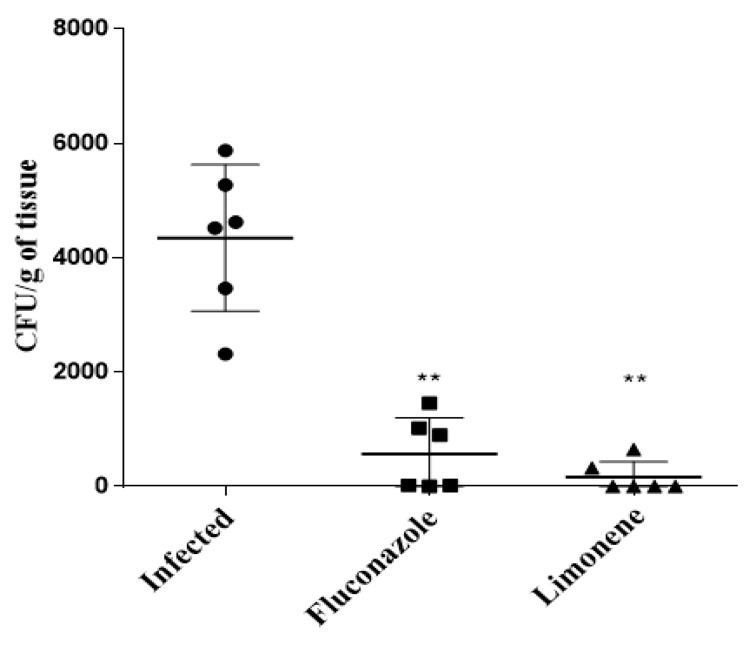
Colony-forming units (CFUs) of the vaginal canal of BALB/c mice that were infected intravaginally with 3 × 10^5^ of *C. albicans* ATCC 90028. Animals (*n* = 6) were treated for 7 days with 10% limonene in cream or with 10 mg/kg of fluconazole in cream and sacrificed after 8 days of infection. The CFUs were quantified after 24 h of growth. ** *p* < 0.01 compared with the control group (*C. albicans* ATCC 90028 without treatment; ANOVA with post-hoc Tukey’s test). Experiments were conducted in three different times.

The number of CFUs in the vaginal canal of mice that received 10% (308.28 µM) limonene was significantly reduced compared to the control (infected but not treated) group. Moreover, we observed a decrease in the number of CFUs in the limonene-treated animals, when compared with the group of animals treated with fluconazole (Figure 4).

### 3.3. Histopathology of the Vaginal canal in Treated, Intravaginally Infected, BALB/c Mice

**Figure 5:**
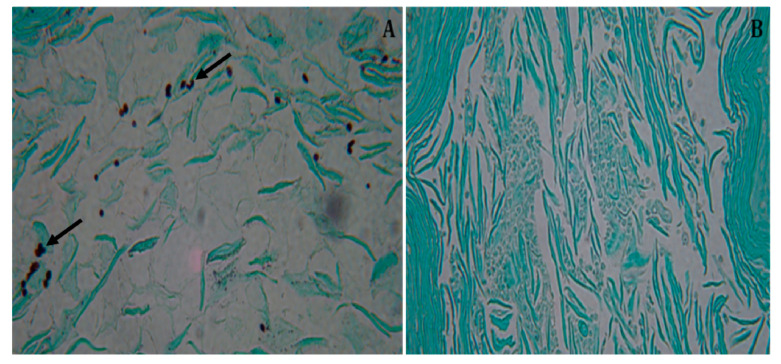
Histopathology of the vaginal canal of BALB/c mice. Histopathology of six BALB/c mice whose vaginal canal was infected with 3 × 10^5^ yeasts of *C. albicans* ATCC 90028. Animals were treated every 24 h, for 7 days, after infection. Treatment was carried out via i.v. (intravaginal) administration, with 10% (308.28 µM) limonene cream. Female mice were sacrificed 8 days after infection. Three vaginal sections per animal were collected and stained with Gomori-Grocott. (**A**) Control group, infected and treated with neutral cream only (arrows indicate the presence of *C. albicans* yeasts on the vaginal canal tissue). (**B**) Vaginal canal of mice treated with 10% (308.28 µM) limonene cream without *C. albicans* yeasts. Original magnification, 40×.

The vaginal canal of the untreated control animals showed multiple yeast-like cells (Figure 5A). In contrast, the animals that received treatment with 10% (308.28 µM) limonene displayed significantly reduced numbers of yeast cells and large areas of preserved vaginal tissue (Figure 5B).

### 3.4. Transmission Electron Microscopy (TEM)

**Figure 6:**
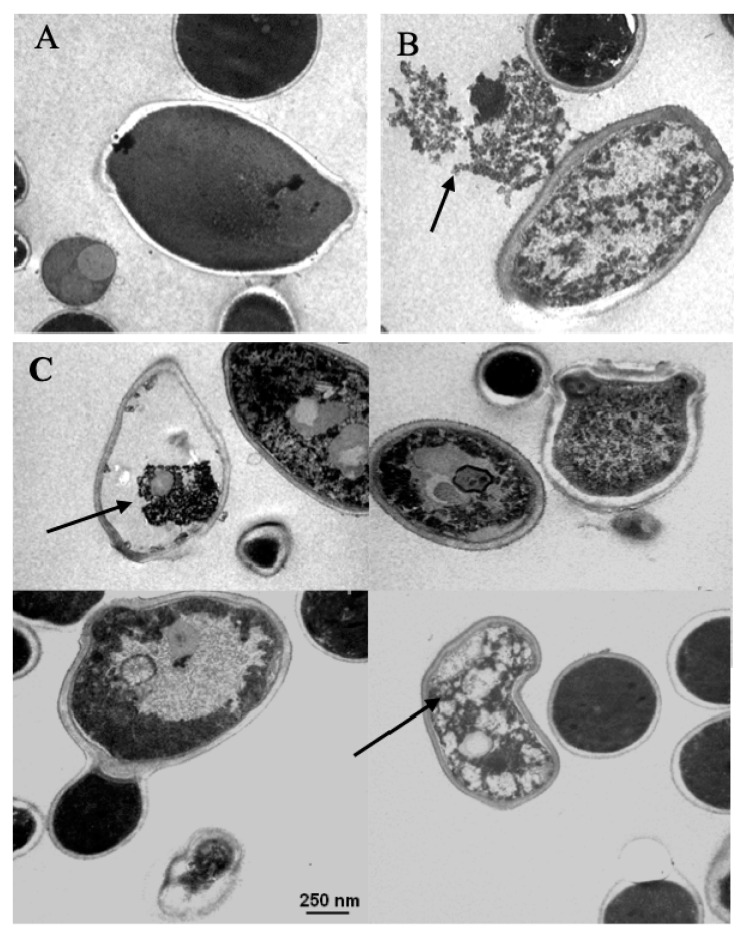
Transmission electron microscopy (TEM) of *Candida albicans* ATCC 90028 yeast. (**A**) Assembly of untreated yeasts, where the total number observed was 36 cells (control group). (**B**,**C**) Assembly of treated yeasts, where the total number observed was 33 cells with 500 µM of limonene. Arrows indicate rupturing of the cell wall and different intracellular disorders probably induced by limonene treatment. Three samples per group were used. Scale bar = 250 nm.

*C. albicans* ATCC 90028 cells treated with different concentrations of limonene displayed a marked decrease in cell density, as fewer cells were detected within the microscope’s field when compared to the untreated controls. A total of 48% of the yeasts treated with 500 µM of limonene showed morphological modifications such as cell wall disruption and significant intracellular damage when compared to the untreated control group (Figure 6). 

### 3.5. Scanning Electron Microscopy (SEM)

**Figure 7:**
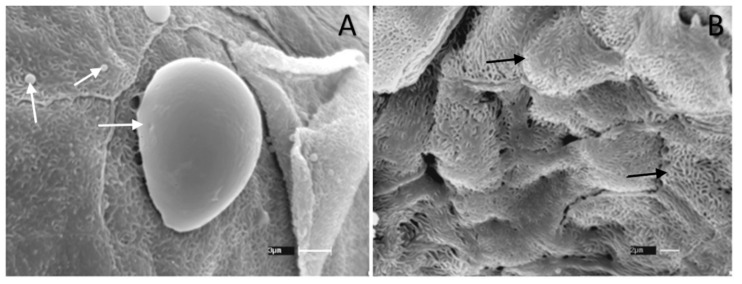
Scanning electron microscopy (SEM) of the vaginal canal from BALB/c mice infected with 3 × 10^5^ yeasts of *C. albicans* ATCC 90028. (**A**) Control group, infected, and treated with neutral cream only. Arrow indicates the presence of *C. albicans* yeasts on the tissue. Scale bar = 3 µm (**B**) Section of vaginal canal from mice treated with 500 µM of limonene cream. Arrows point to the squamous cells present in the epithelium. Scale bar = 2 µm. Three samples per group were analyzed.

Yeast cells could be easily detected by SEM in the vaginal canal of animals inoculated with *C. albicans* without limonene treatment (Figure 7A), but not in the vaginal canal of animals infected and treated with 500 µM of limonene. However, we observed some epithelial irritation signs, as evidenced by the presence of squamous cells (Figure 7B).

## 4. Discussion

In the present work, we evaluated the antifungal activity of R-(+)-limonene against clinical and ATCC isolates of *Candida* spp. susceptible or resistant to antifungal drugs. R-(+)-limonene is largely used in food, beverage, and cosmetic industries and is classified as a low-toxicity additive [27]. The efficacy of limonene as an anti-diabetic, anticarcinogenic, and antimicrobial agent has also been evaluated with relative success [27].

In vivo and preclinical models identified that the oral administration of R-(+)-limonene was absorbed from the rat gastrointestinal tract by diffusion (reviewed by Chandrakala et al., 2018) and also by humans with little toxicity [28]. These data are important considering the small amount of R-(+)-limonene used here in the intravaginal treatment compared with the oral treatment. Future studies may be carried out to analyze the possible absorption of R-(+)-limonene by the vaginal mucous.

To define the best concentration of R-(+)-limonene able to interfere with the growth of yeast cells, a kinetic analysis was performed and our results confirmed that limonene at concentrations ≥500 µM can inhibit the growth of *C. albicans*, *C. krusei*, *C. glabrata*, and *C. parapsilosis* in vitro, reinforcing previous observations made by Freire and collaborators (2017), who originally evaluated the antimicrobial properties of limonene-containing essential oils, derived from *Mentha piperita L.*, *Origanum vulgare*, and *Zingiber officinalepara* [29]. Interestingly, all these oils were capable of exerting a fungicidal effect on *Candida* strains (obtained from dental prostheses), inducing morphological alterations in yeast cells and inhibiting pseudohyphae formation [29]. Similarly, Leite and coworkers (2014) detected that *C. albicans* yeasts treated with citrus essential oils, containing several substances structurally related to limonene, also displayed morphological alterations and decreased chlamydoconidia production, which directly affected the pathogenicity of the treated yeasts [30].

In addition to inhibiting growth, we assessed the ability to interfere with the viability of yeast. We observed that the viability of the yeasts treated with limonene in concentrations ≥500 µM was significantly reduced. Previous studies described that the use of this monoterpene in concentrations between 0.6 to 5 mM induces apoptosis in *C. albicans*, due to the damage caused to both the cell wall and membrane. In that study, the authors claimed that cell death was likely due to oxidative stress produced by limonene, which leads to DNA damage, alterations of the cell cycle, and, finally, yeast apoptosis [31].

Mondello and collaborators (2003) employed a rat vulvovaginal candidiasis model to demonstrate that the essential oils of *Malaleuca alternifolia* (composed of a complex mixture of monoterpenes) were protective against *C. albicans* strains either susceptible or resistant to fluconazole and/or itraconazole [32]. Cases of recurrent vulvovaginal candidiasis, mainly caused by *C. albicans*, have drawn particular attention from the medical community over the past years, given their high prevalence and challenging treatment. It has been described that the difficulty in treating this mycosis is directly associated with a variety of factors, including the presence of drug-resistant *Candida* strains (mostly non-*albicans* species), overall predisposition of patients to infection, use of antibiotics, uncontrolled diabetes, estrogen use, pregnancy, a host’s genetic factors, and alterations in the vaginal microbiota, among others [33,34,35].

To the best of our knowledge, the present work represents the first example of a successful R-(+)-limonene-based treatment of vulvovaginal candidiasis in mice. Moreover, our studies lead us to verify that limonene also exerts a protective role against *C. albicans* infections, since animals that were submitted to intravaginal limonene treatments showed a significant decrease in the fungal burden when compared to untreated animals. 

Transmission electron microscopy studies performed on *Candida* yeasts showed damage to the cell wall, as well as to intracellular structures, including nuclear alterations, such as the condensation of genetic material and specific changes in mitochondria. Moreover, yeasts treated with limonene also displayed dramatic structural changes in organelles, accompanied by cell wall rupture in most of the observed fields. These alterations may have derived from the action of limonene on the yeast genetic material, as described by Thakre and collaborators (2018). Recent work has also demonstrated that monoterpenes can interfere with the permeability and fluidity of plasma membranes and can inhibit efflux pumps, possibly creating permanent membrane pores, resulting in cytoplasmic leakage and irreversible cellular damage [36].

Finally, scanning electron microscopy analyses showed distinct alterations in the appearance of the vulvovaginal canal epithelium, possibly due to an irritation process. This observation conflicts with observations made by Arruda and coworkers (2009), who carried out cytotoxic assays using R-(+)-limonene at concentrations reaching up to 1.012 μM, which resulted in no observable cytotoxic effect on monkey epithelial cells (LLCMK2) or human cells (HEK-293). Another important aspect is that R-(+)-limonene did not induce mutagenic, carcinogenic, or nephrotoxic effects in humans [37]. In mice, the oral LD50 for limonene was reported to be 5.6 g/kg body weight in males and 6.6 g/kg body weight in females [37]. 

Since vulvovaginal candidiasis is an infection that affects up to 70% of all women, at least once during their reproductive years, and given the fact that approximately 10% of these cases may turn into recurrent vulvovaginal candidiasis, it is important to search for new therapeutic alternatives to treat this pathology [33]. In this sense, the present study strongly suggests that topical R-(+)-limonene has significant therapeutic benefits in experimental vulvovaginal candidiasis.
